# Atomic-scale insights on the formation of ordered arrays of edge dislocations in Ge/Si(001) films via molecular dynamics simulations

**DOI:** 10.1038/s41598-022-07206-3

**Published:** 2022-02-25

**Authors:** Luca Barbisan, Anna Marzegalli, Francesco Montalenti

**Affiliations:** 1 Department of Materials Science, L-Ness and Università degli Studi di Milano-Bicocca, Via R. Cozzi 55, 20125 Milan, Italy; 2 Department of Physics, L-Ness and Politecnico di Milano, via Anzani 42, 22100 Como, Italy

**Keywords:** Semiconductors, Atomistic models, Structural properties, Condensed-matter physics, Nanoscale materials

## Abstract

Heteroepitaxial films of Ge on Si(001) are receiving wide attention due to several possible applications in micro- and opto-electronics. Understanding the dynamic behavior of linear defects, such as dislocations, is key. They are unavoidably present in such systems due to the lattice mismatch between the two materials, and can directly influence devices performances. It has been experimentally demonstrated more than fifteen years ago that a suitable choice of the growth parameters allows for the formation of a nicely ordered net of $90^{\circ }$ dislocations at the Ge/Si interface, improving the overall film quality and strain relaxation uniformity. Atomic-scale details on the set of mechanisms leading to such an outcome are however still missing. Here we present a set of classical molecular dynamics simulations shedding light on the full set of microscopic processes driving to the experimentally observed array of linear defects. This includes simple gliding of $60^{\circ }$ dislocations and vacancy-promoted climbing and gliding. The importance of the particular experimental conditions, involving a low-temperature stage followed by an increase in temperature, is highlighted.

## Introduction

Due to its compatibility with standard silicon (Si) technology and to several superior properties with respect to Si, Germanium (Ge) has attracted widespread attention. We recall that Ge displays high carrier mobility, a small difference between its direct and indirect band gaps, and a strong absorption band at near-infrared wavelengths^1–5^. Unsurprisingly then, Ge/Si(001) heteroepitaxial films are appealing for optoelectronic applications such as light emission in integrated circuits, as channel materials in field-effect transistors^6–9^, and high-speed optoelectronic devices^10–12^. Moreover, because of the small difference between the gaps in $\Gamma$ and *L* points, the band-related characteristics can be tuned via strain engineering as well as a further improvement of carriers mobility^13–15^.

Integration of Ge/Si(001) is not straightforward due to the large lattice mismatch between film and substrate (+4.2%). If growth conditions close to thermodynamic equilibrium are maintained, the system follows the Stransky–Krastanow growth modality with three-dimensional islands appearing after the formation of a wetting layer^16–18^.

As three-dimensional growth, allowing for partial relaxation of the lattice mismatch^16,19,20^, is unsuitable for most applications, out-of-equilibrium conditions are typically exploited to grow flat films. These can be achieved by lowering the growth temperature and/or by using high deposition rates^21–24^. Under such conditions, the lattice mismatch is solely released via plastic relaxation following dislocation nucleation. Often (see, e.g.^21,22^) the temperature is kept low only in a first phase, while increased to improve the film quality at a second stage, when the strain in the system is sufficiently relieved by defects to avoid island formation^25^.

In Ge/Si(001) epitaxial heterostructures, plastic relaxation occurs mainly through the nucleation at the surface of dislocation (semi-)loops^26^, with Burgers vectors $\vec{b} = a/2\langle 011\rangle$—*a* being the epilayer lattice parameter—gliding in $\{111\}$ planes. As the loop radius increases the dislocation progressively releases the epi-layer lattice compression, eventually creating a 60$^{\circ }$ misfit dislocation segment at the hetero-interface. Experiments evidenced also the presence of edge dislocations at the interface, with their density increasing at the expense of 60$^{\circ }$ dislocations when a high temperature or annealing step follows the low-temperature deposition^21–24,27^. Actually, it was clearly demonstrated that thermal treatment in the form of annealing leads to a formation of an ordered network of 90$^{\circ }$ (edge) dislocation segments at the interface, improving film quality and strain relaxation uniformity^28,29^. Edge misfit dislocations (often called Lomer dislocations) are characterized by Burgers vectors $\vec{b} = \pm a/2[1{\bar{1}}0]$ or $\vec{b} = \pm a/2[110]$ lying in the interface plane. Two different questions are still under discussion: (i) How are 90$^{\circ }$ dislocations formed? (ii) How is lateral ordering of dislocation at the interface reached?

A partial answer to the first question was supplied as different possible mechanisms leading to the formation of edge dislocations at the interface were proposed^30,31^. Experiments and theoretical calculations^31–34^ have highlighted the importance of one of them, i.e. preferential nucleation of a “complementary”^35^ 60$^{\circ }$ dislocation: the stress field induced in the proximity of the free surface by the presence of a 60$^{\circ }$ dislocation at the Ge/Si interface lowers the barrier for nucleation of a second (the “complementary one”) 60$^{\circ }$ dislocation with Burgers vector such that the superimposition of the two generates a 90$^{\circ }$ dislocation. In order for this reaction to take place through simple dislocation gliding, however, the original dislocation must lay on the glide plane of the complementary one. While this can happen in some cases, the most probable configuration leads to close pairs of 60$^{\circ }$ dislocations, clearly observed in low-temperature experiments^27,32^. How such pairs evolve into a 90$^{\circ }$ dislocation upon annealing is not yet clarified. Similarly, no clear answer to question (ii)—regarding annealing-induced ordering—is present in the literature. The possible role of vacancies in influencing gliding/climbing and lateral ordering was invoked^21^ but only at the speculative level. As reaching defect uniformity is an important requirement for applications, we find it of utmost importance to provide a solid atomic-scale explanation.

In this work we shall clarify both the mechanism of 90$^{\circ }$ dislocation formation from pairs of complementary 60$^{\circ }$ dislocations and of lateral ordering at the Ge/Si interface by suitable molecular dynamics (MD) simulations, highlighting the key role played by vacancies in influencing defects motion and the importance of the growth/post-growth conditions.

## Methods

All simulation results presented in this work were obtained in the framework of classical molecular dynamics as implemented in the Large-scale Atomic/Molecular Massively Parallel Simulator (LAMMPS)^36^. The Tersoff potential^37^ was used to describe interatomic interactions. While obviously not exact, such an approach has been widely exploited to model defects in Ge/Si systems^32,38–40^, yielding results compatible with experimental evidence. Simulations presented in the present article were run in the canonical ensemble, using a Nose–Hoover thermostat^41^ and setting the time step to 1 fs. The latter was calibrated in a set of dedicated microcanonical simulations where relative fluctuations in total energy lower than $10^{-5}$ were observed even at the highest ($T={1400}\,\hbox {K}$) sampled temperature.

Orthogonal simulation cells, oriented along the directions determined by the vectors $x=[{\bar{1}}10]$, $y=[110]$, $z=[001]$ were used, and periodic boundary conditions (PBCs) were applied in the *x* and *y* directions. To model a strained epitaxial layer, we created a simulation cell composed of 32 monolayers of Ge atoms on top of other 32 monolayers of silicon atoms along the $z=[001]$ direction. The lattice parameter was set as the one of pure silicon. In this way germanium atoms were strained in the *x*, *y* directions $\varepsilon _{xx}=\varepsilon _{yy}\approx 4\%$. To simulate the effect of a thick bulk substrate below the Ge layer, the bottom three layers of silicon atoms in the simulation cell were kept fixed to bulk positions. The top Ge surface was reconstructed $2\times 1$ upon forming dimers accordingly. We performed simulations of dislocation arrays by using three different cell dimensions ($9.987/14.980/19.973\times 1.536\times 8.530\;\hbox {nm}^{3}$) depending on the explored strain conditions. The cells are made up by replicating the perfect diamond lattice unit cell $26/39/52\times 4\times 16$ times.

Atomic trajectories were analyzed using the Open Visualization Tool (OVITO) software^42^, which allows for a very convenient identification of defects in crystals, e.g., providing dislocation lines along with the associated Burgers vectors.

### Dislocation modeling

For the sake of simplicity, dislocations were introduced in the system as straight segments running along the *y* direction. Starting from a more realistic (half)loop configuration would have required a larger cell in the *y* direction, making it impossible to reach the remarkable time scales simulated when describing climb (see the “Results” section). Instead, we could set the cell length along *y* as relatively small (${1.536}\,\hbox {nm}$). Shortening the dislocation line further would be critical as it would preclude the formation of kinks and jogs, allowing for the natural motion of dislocations. On the other hand, despite being minimal, we verified that the simulation cell was indeed large enough along *y*: we repeated a subset of the simulations using a three-time larger cell along *y* without observing any significant dependence of the results on the dimensions (see Supplementary Information online).

Dislocation lines were inserted in the simulation cell by shifting each atom by the displacement field vectors calculated by dislocation modeling in the framework of the linear elasticity theory^43^. The obtained configurations were geometrically optimized using a conjugated gradient minimization algorithm. We recall that, for an arbitrary dislocation segment, the field components can be easily calculated by rotating the reference system and decomposing the dislocation Burgers vector into a screw ($b_{\parallel }$) and an edge component ($b_{\perp }$). At this, ${\hat{z}}$ axis has to be oriented along the dislocation line $\xi$, ${\hat{x}}$ axis perpendicular to $\mathbf {\xi }$ in the glide plane, and ${\hat{y}}$ axis oriented following the right hand rule, respectively. In this case, the components of the displacement field vectors are written as follows:

1a $$\begin{aligned} u_x(x,y,z)&=\,\, \frac{b_\perp }{2\pi }\left( \mathrm {arctan} (y/x)+\frac{x\,y}{2(1-\nu )(x^2+y^2)}\right) \end{aligned}$$

1b $$\begin{aligned} u_y(x,y,z)&=-\frac{b_\perp }{2\pi }\left( \frac{(1-2\nu )\mathrm {log} (x^2+y^2)}{4(1-\nu )}+\frac{x^2-y^2}{4(1-\nu )(x^2+y^2)}\right) \end{aligned}$$

1c $$\begin{aligned} u_z(x,y,z)&=\,\,\frac{b_\parallel }{2\pi }\,\mathrm {arctan} (y/x) \end{aligned}$$

where $\nu$ = 0.26 is the Poisson ratio of Ge^26^. Equation (1) gives the displacement field associated with an isolated dislocation in bulk. Before applying it to the atoms in the simulation cell we found it convenient to also add the contribution of the image dislocation^43^, introduced by the presence of the free surface. We also added the additional contribution induced by the presence of PBCs along the *x* direction^44^ creating a dislocation array out of a single defect.

### Simulation of vacancy-induced dislocation motion

Dislocation climbing requires vacancies to migrate to the dislocation core along the whole dislocation line^43^. Brute-force runs, where both typical experimental temperatures and physically-sound vacancy concentrations are considered, do not show any significant evolution due to the timescale limitations imposed by MD. Even if one disregards experimental conditions, observing climbing along the full dislocation line with MD is not trivial. One can attempt to speed up the motion by increasing the simulation temperature, raising the vacancies’ diffusion coefficient. However, there exists a limit beyond which excessive disorder is introduced in the system (we used up to $T={1400}\,\hbox {K}$, high but well below the Tersoff-potential Ge melting temperature^45^). Instead of manipulating the diffusion coefficient, other approaches can be used. For instance, Li et al. pre-positioned an entire row of vacancies on the dislocation path, therefore forcing climbing motion^46^ once the dislocation reached them. A step forward would require to simulate also the motion of vacancies towards the dislocations. To achieve this within reasonable computational time, it is required to reduce the path a vacancy must travel to reach the dislocation core. A possible way to accomplish this is to consider very high vacancy densities. If vacancies are placed too close to each other, however, the formation of slowly-diffusing vacancy complexes precludes observation of migration to the dislocation core. After attempting several strategies along the lines traced here-above, we came up with a procedure that we find particularly appealing since the system is left sufficiently free to evolve, and climbing of the entire dislocation line can be observed. The procedure is the following one: we start by introducing a dislocation (an extension to two dislocations is also exploited in the “Results” section) in the simulation cell. We then remove one atom of the crystal randomly. We start the MD simulation and track the vacancy motion on the fly by performing a Wigner Seitz Defect Analysis using the OVITO software. If the vacancy ends up at the dislocation core or at the top free surface, we then randomly insert a second vacancy and start tracking its motion. Otherwise, we keep on simulating the system dynamics. The procedure is iterated until the desired number of vacancies are inserted. As demonstrated in the “Results” section, the procedure works and allows to observe the vacancy-induced motion of a full dislocation line.

In principle, we could have randomly created vacancies within the whole simulation cell. However, to decrease the time needed for the vacancy to reach the dislocation core, we defined a smaller “vacancy-spawn” region—where the point defect was created- composed of the atoms distant less than ${3}\,\hbox { nm}$ from the dislocation core. The 3 nm size of the “vacancy-spawn” region is relevant only for speeding up the simulations. Considering a “spawn-region” large as the whole cell along *x* did not change the ratio of vacancies that effectively migrate to the dislocation core. In addition, such a region was restricted to be at a distance from the upper free surface larger than the one of the dislocation core as vacancies created between the dislocation core and the free surface were observed to annihilate at the latter irreversibly.

## Results and discussion

### Gliding of pairs of 60$^{\circ }$ misfit dislocation

**Figure 1:**
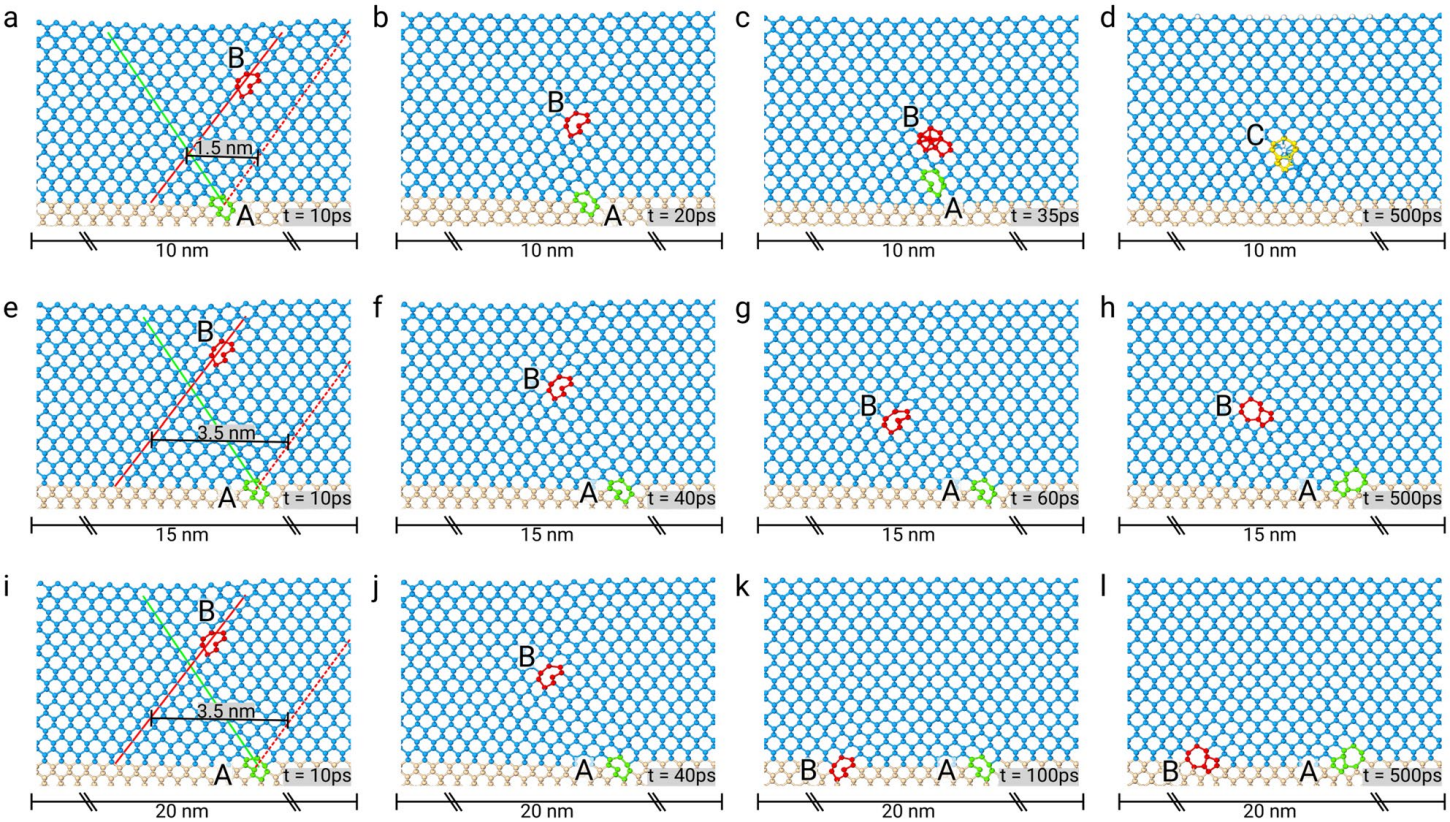
Evolution of complementary dislocations pairs. Each row corresponds to a different simulation performed using different cell sizes to simulate different degrees of relaxation. Light blue spheres represent Ge atoms, pink spheres Si atoms, green spheres highlight the core of the dislocation “A,” which starts at the interface (Burgers vector $\vec{b}=a/2[011]$), red spheres highlight the core of the complementary dislocation “B”, ${1.5}\,\hbox {nm}$ below the free surface (Burgers vector $\vec{b}=a/2[{\bar{1}}0{\bar{1}}]$). The glide planes of the dislocations are shown in the first row by colored solid lines; the mirror-like glide plane is shown as a dashed line. (**a**–**d**) Complementary 60$^{\circ }$ dislocations joining above the interface forming the pure edge 90$^{\circ }$ dislocation “C”; the glide plane of the second dislocation is ${1.5}\,\hbox {nm}$ away from the mirror-like glide plane, and the dimension of the simulated cell is ${10}\,\hbox {nm}$ in the direction perpendicular to dislocation lines. (**e**–**h**) Complementary 60$^{\circ }$ dislocation blocked above the interface; the glide plane of the second dislocation is ${3.5}\,\hbox {nm}$ away from the mirror-like glide plane, and the dimension of the simulated cell is ${15}\,\hbox {nm}$ in the direction perpendicular to dislocation lines. (**i**–**l**) Complementary 60$^{\circ }$ dislocations both at the interface; the glide plane of the second dislocation is ${3.5}\,\hbox {nm}$ away from the mirror-like glide plane, and the dimension of the simulated cell is ${20}\,\hbox {nm}$ in the direction perpendicular to dislocation lines.

As already stressed in the introduction, induced nucleation of a second complementary 60$^{\circ }$ dislocation by a first 60$^{\circ }$ dislocation at the Ge/Si interface has been recognized as the primary cause for the creation of 90$^{\circ }$ dislocation and of 60$^{\circ }$ dislocation pairs^32,47^. As no atomic-scale description of further evolution under annealing is present in the literature, we shall start our analysis precisely from these pairs. Following Ref.^32^, the typical initial condition leading to their formation is given by having one 60$^{\circ }$ dislocation—called “A” in Fig. 1—at the Ge/Si interface and a second one—“B” in Fig. 1—nucleated close to the free surface. If the position of the core of dislocation “A” belongs to the glide plane of dislocation “B” (this glide plane is often called mirror glide plane, and is indicated by a red dashed line in Fig. 1) then simple gliding of “B” towards “A” can lead to the formation of a 90$^{\circ }$ dislocation. For this case, annealing is not needed to promote the existence of such a defect. A close pair of 60$^{\circ }$ dislocations is formed when, instead, dislocation “B” glide plane intersects dislocation “A” glide plane not at the interface. Three representative initial configurations are displayed in Fig. 1, together with the evolution predicted by molecular dynamics simulations at ${1000}\,\hbox {K}$. Dislocation “A”, with Burgers vector $\vec{b}=a/2[011]$ is placed at the interface (the dislocation core is highlighted in green), dislocation “B”, with Burgers vector $\vec{b}=a/2[{\bar{1}}0{\bar{1}}]$, is placed ${1.5}\,\hbox {nm}$ below the free surface (the dislocation core is highlighted in red). The glide planes of the two dislocations are misaligned (they do not intersect at the interface); therefore, they cannot form a 90$^{\circ }$ dislocation at the interface. The distance $d_{ML}$, between the glide plane of the second dislocation (at the interface) and the mirror-like glide plane, is used as a parameter. Three cell sizes along the *x* direction were used to simulate different relaxation of the Ge film: $\approx$ ${10}\,\hbox {nm}$—corresponding to full relaxation of the $\sim 4\%$ Ge/Si lattice mismatch in the presence of a 90$^{\circ }$ edge dislocation at the interface per cell, $\approx$ ${15}\,\hbox {nm}$—i.e., 75% relaxation with a 90$^{\circ }$ edge dislocation at the interface, $\approx$ ${20}\,\hbox {nm}$—i.e., half relaxation with a 90$^{\circ }$ edge dislocation at the interface.

**Figure 2:**
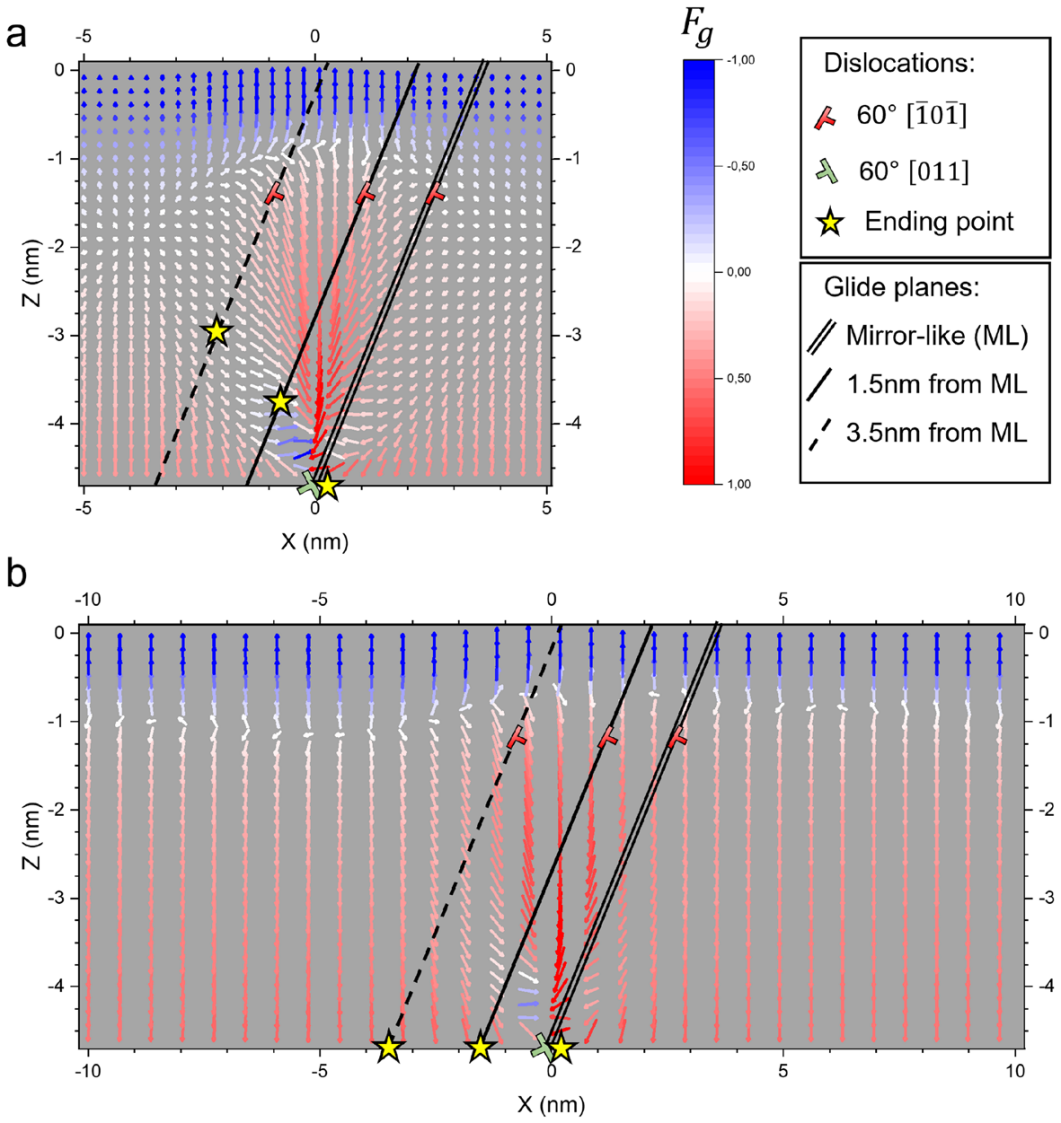
Map of the Peach and Koehler force acting on dislocation “B” with Burgers vector $\vec{b}=a/2[{\bar{1}}0{\bar{1}}]$ as a function of its position in a Ge film. The complementary dislocation “A” with Burgers vector $\vec{b}=a/2[011]$ is fixed at the interface in $x=0$. The total (i.e., glide and climb together) force vectors are colored in blue-red scale to represent the projection of the Peach and Koehler force along the glide plane. Black lines are inserted to show some possible glide planes of dislocation “B”: double line—mirror-like glide plane, solid line—glide plane at $d_{ML}={1.5}\,\hbox {nm}$, dashed line—glide plane at $d_{ML}={3.5}\,\hbox {nm}$. For each glide plane, a star indicates the position where the dislocation will stop following the force gradient. Panel (**a**) cell size ${10}\,\hbox {nm}$, panel (**b**) cell size ${20}\,\hbox {nm}$.

A wide range of relative arrangements of two complementary dislocations are possible and was observed in low-T experiments. Still, the results of their evolution can be grouped in a few categories only: an edge dislocation at the Si/Ge interface (see Brunetto et al.^32^), an edge dislocation few nanometers above the Si/Ge interface, a 60$^{\circ }$ dislocation “floating” few nanometers above the Si/Ge interface in the vicinity of its complementary counterpart, and two complementary 60$^{\circ }$ dislocations both at the interface. MD simulations were able to fully capture all these behavior, as seen in Fig. 1. Indeed, different initial positions of the complementary dislocation placed at the surface and different stress in the cell (i.e., different cell size) lead to the configurations experimentally observed. The evolution reported in panels Fig. 1a–d ends with a 90$^{\circ }$ dislocation above the interface, the ones in Fig. 1e–h with the stopping of the complementary dislocation within the layer, and that in Fig. 1i–l with both the dislocation at the interface.

To explain the results of the here-above reported molecular dynamics simulations, we find it helpful to show, in Fig. 2, the force field experienced by dislocation “B” placed in different positions while keeping dislocation “A” at the Ge/Si interface. Such force field can be conveniently computed based on elasticity theory^48^, including periodic boundary conditions and the effect of the top free surface. Figure 2 allows for an immediate interpretation of the various configurations obtained at the end of the molecular dynamics simulations of Fig. 1. Note that, differently from molecular dynamics, the analytical calculation assumes^48^ an isotropic medium. However, it is evident from the comparison between Fig. 2 and the final configurations of Fig. 1 that the qualitative behavior found by MD and predicted by the analytical model corresponds.

The force field displayed in Fig. 2a is computed for an array of dislocations with a periodicity of ${10}\,\hbox {nm}$ (ideal periodicity of 90$^{\circ }$ dislocations to relax pure Ge on Si). The one shown in Fig. 2b, instead, corresponds to a ${20}\,\hbox {nm}$ periodicity. The force vectors are colored in red/blue to show the intensity and the orientation of the glide component of the force in a specific position. A saturated red means a strong glide force towards the Si/Ge interface, saturated blue means a strong glide force towards the free surface, pale/white hues indicate a small/zero force. In Fig. 2 a star indicates where dislocation “B” will stop its glide for some selected glide planes. The formation of an edge dislocation at the Si/Ge interface is achieved when dislocation “B” nucleates and glide along a plane which is exactly the mirror-like glide plane (double solid line in Fig. 2). In this case, the force acting on the dislocation is almost aligned to the glide plane resulting in an easy glide of the dislocation. An edge dislocation above the Si/Ge interface can be formed when dislocation “B” nucleates slightly apart from the mirror-like glide plane. Consider for reference the single solid line in Fig. 2; in this case, dislocation “B” can move on a glide plane which is ${1.5}\,\hbox {nm}$ away from the mirror-like glide plane. The projection of the force acting on dislocation “B” on its glide plane (i.e., the glide component of the force) changes in sign a few nanometers above the Si/Ge interface (see red/blue color-changing). Therefore the dislocation, in this case, will glide towards the Si but will stop in the vicinity of its complementary dislocation (a star indicates the position where the dislocation will stop in Fig. 2). It clearly corresponds to what happen in the molecular dynamics simulation of Fig. 1a–d. At that height dislocation “B” can attract dislocation “A”, making it gliding upward to merge and form a 90$^{\circ }$ dislocation at their junction (Fig.1d).

A qualitatively different result is obtained by placing dislocation “B” further away from the mirror-like glide plane. Indeed, when the distance between the glide plane of “B” and the mirror-like glide plane ($d_{ML}$) is ${3.5}\,\hbox {nm}$ (dashed black line in the Fig. 2a) we can notice that at a certain height the force acting on the dislocation is directed for the most outside its glide plane. This can result in an evolution which is analogous to that reproduced via molecular dynamics and shown in Fig. 1e–h: dislocation “B” migrate from the free surface towards the Si/Ge interface, but in the middle of the film it stops in a local minimum position because of the small glide component of the Peach and Koehler force. Dislocation “B” remains floating in the Ge film, too far away from dislocation “A” to make it glide towards it.

This behavior can be expected to change in the case of a much higher strain. Indeed using a periodicity of ${20}\,\hbox {nm}$ instead of ${10}\,\hbox {nm}$, as shown in Fig. 2b, the glide component of the Peach and Koehler force is much higher, and dislocation “B” is pushed towards the Si/Ge interface with almost the same force intensity throughout all the Ge film. In this latter case dislocation “B” will reach the interface, exactly as reproduced via the molecular dynamics simulations illustrated in Fig. 1i–l. We have checked that in the intermediate strain condition (${15}\,\hbox {nm}$ periodicity), the final dislocation configuration is similar to those reported in the ${10}\,\hbox {nm}$ map.

In many low-temperature growth experiments, proofs of the presence of these particular arrangements of complementary dislocations are commonly observed^27,32^, but when the successive annealing at high temperature is performed, most of the defects are found at the interface, they are ordered, and most of them are 90$^{\circ }$ edge dislocations^21,27,29^. The pure glide motion of 60$^{\circ }$ misfit dislocations cannot explain either the formation of 90$^{\circ }$ misfit dislocation to a large extent or the ordering of the 90$^{\circ }$ dislocation arrays. Climbing processes are often evoked to explain that experimental evidence, but no atomic-scale description based on simulations was given. Here we present our analysis filling the gap.

### Climbing of 90$^{\circ }$ misfit dislocation

**Figure 3:**
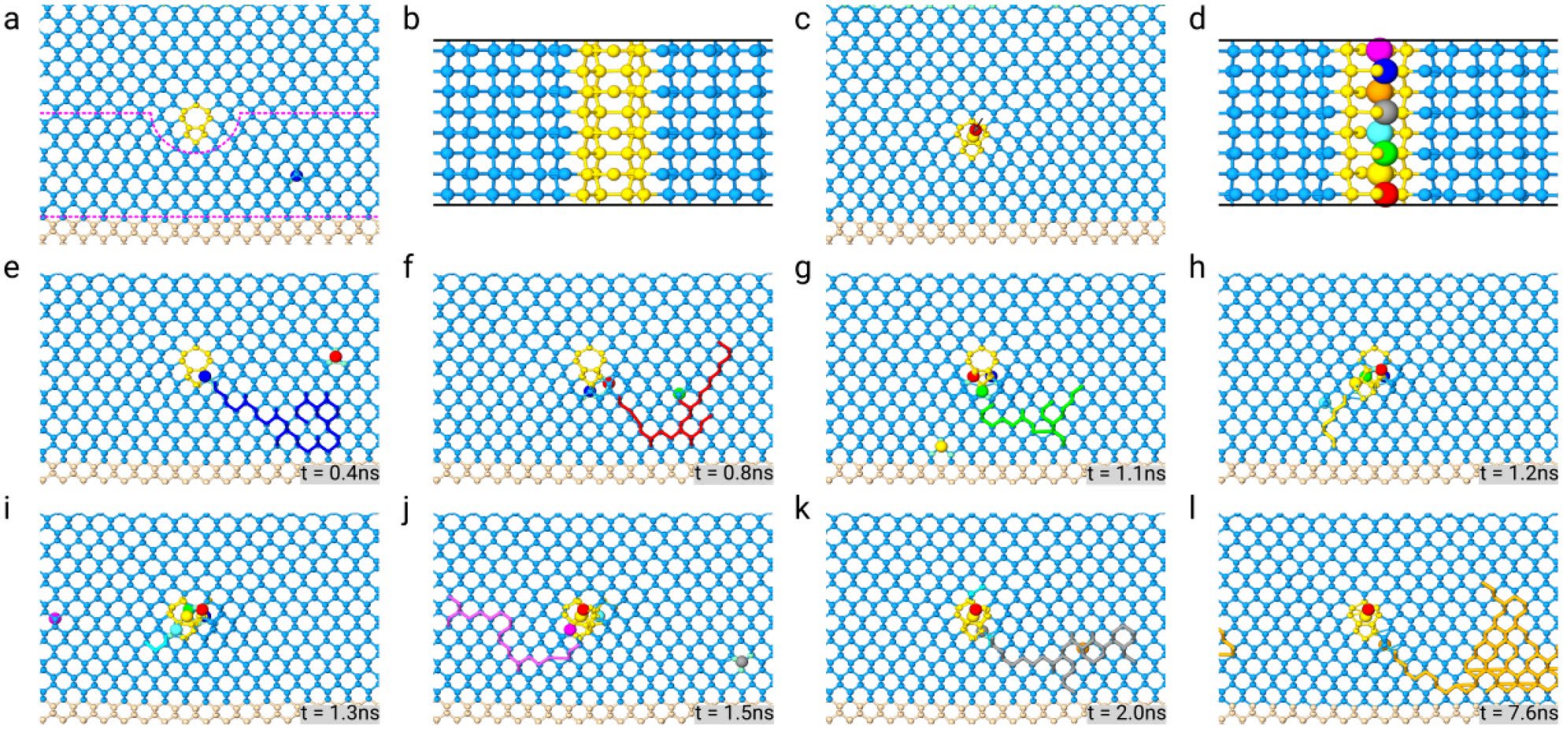
Simulation of edge dislocation climb. Light blue spheres represent Ge atoms, pink spheres Si atoms, yellow spheres highlight the core of an edge dislocation ${2.0}\,\hbox {nm}$ above the interface (Burgers vector $\vec{b}=a/2[{\bar{1}}10]$). Larger spheres show the position of the vacancies as identified by Wigner–Seitz defect analysis in OVITO. A trajectory is provided for each vacancy by a colored line. (**a**) Starting configuration: a Lomer dislocation ${2.0}\,\hbox {nm}$ above the Si/Ge interface, the “vacancy spawn” region is shown by the dashed purple line. (**b**) top view of the dislocation core. (**c**) final snapshot after eight vacancies has been inserted in the cell (a black arrow shows the displacement of the dislocation), with the corresponding top view (**d**). Simulation snapshots are provided in sequence; the corresponding simulation time is shown in the inset (**e**–**l**).

We analyzed the role of the climbing mechanism in the formation and ordering of 90$^{\circ }$ edge dislocations starting from the configuration found as the final state reached by glide motion. In particular, we first considered the configuration of dislocations in Fig. 1d—i.e. a 90$^{\circ }$ Lomer dislocation formed above the Si/Ge interface. Climbing of an edge dislocation is the simplest case of dislocation climbing motion and is often used in the literature as a prototypical example^43^. We have already described in the “Methods” section how we triggered climbing by inserting vacancies one at the time, removing atoms of the crystal randomly within a “vacancy spawn region,” before simulating the system evolution at $T={1400}\,\hbox {K}$ and checking whether the conditions to insert a new vacancy are met. Here we shall illustrate the procedure further by analyzing the actual simulation where climbing of the 90$^{\circ }$ edge dislocation is tackled (Fig. 3). As shown in Fig. 3e, the first vacancy, which is represented by the large blue sphere, is observed to diffuse through the Ge film (blue trajectory in the same Figure) while the edge dislocation stays immobile. After few hundred ps, the vacancy reaches the dislocation core. At this point another vacancy is inserted (red sphere in Fig. 3e) and the system is evolved until also the second vacancy reaches the dislocation core (see Fig. 3f, this time its trajectory is highlighted in red). The simulation is iterated, and a new vacancy is created in the “vacancy spawn” region every time there are no vacancies in that region, i.e., the vacancy has reached the dislocation core or is lost towards the free surface. The third vacancy (green sphere) reaches in turn the dislocation core (Fig. 3g), and thereafter, in few hundreds of ps, is followed by the fourth vacancy (yellow sphere, Fig. 3h). With four vacancies, there is a section of the dislocation line that is one layer below its original position, i.e., a jog has formed. A fifth vacancy is inserted and it migrate to the dislocation core (tile sphere, Fig. 3i). The successive vacancy is the sixth one, and it also reaches the dislocation core that now is almost completely one glide plane below its starting position (purple sphere, Fig. 3j). The seventh vacancy (gray sphere, Fig. 3k) does the same, and finally the last vacancy migrates to the dislocation core, achieving the complete climb of the dislocation (orange sphere, Fig. 3l). The starting and final configuration are shown them Fig. 3a, c, respectively. A black arrow show the displacement of the dislocation core from its starting position. The top views of the dislocation core with and without vacancies are shown in Fig. 3d, b, respectively.

The analysis of vacancy trajectories clearly shows that the motion of a single vacancy is random-like at the beginning, while in the proximity of the dislocation core, the attraction between the point and the linear defect leads to the irreversible capture of the former at the core of the latter. A video animation of the climb of an edge dislocation on a three times larger cell can be found as Supplementary Video S1 and Supplementary Fig. S1 online. It is also evident that vacancies are attracted by the compressive deformation below the dislocation core. Indeed, a relevant trend, which can be easily observed by considering trajectory lines, is that once vacancies are close to the dislocation core, they start to move towards the dislocation preferentially along {111} planes. Note that the dislocation’s climb movement turns to be a step towards the interface along a $\langle 111\rangle$ direction.

### Climbing motion involving pairs of 60$^{\circ }$ misfit dislocation

The configurations that we have found by pure gliding (Fig. 1) and considered for the formation of purely 90$^{\circ }$ edge dislocation at the interface were grouped into three classes. The first set was made up by an edge dislocation a few nanometers above the interface, and its evolution was described in the preceding section. Then two other sets were possible, both composed of complementary 60$^{\circ }$ misfit dislocation pairs. Being clarified that, when we follow the evolution from the first configuration, 90$^{\circ }$ dislocation can be found at the Si/Ge interface because of climbing motion activated by vacancies; it remains to be understood if the same stems for pairs of complementary 60$^{\circ }$ misfit dislocations. Two configurations are possible: two 60$^{\circ }$ complementary dislocations spatially separated at the interface (see Fig. 1i–l for reference) or a 60$^{\circ }$ dislocation blocked in the film above the interface in the vicinity of its complementary pair (such as in Fig. 1e–h). After having demonstrated that the introduction of one vacancy at a time in the simulation cell allows one to conveniently simulate climbing of an edge dislocation, we extended the same procedure for investigating vacancy-induced motion of 60$^{\circ }$ pairs. In particular, we started by investigating the evolution of a 60$^{\circ }$ dislocation blocked a few nanometers above its complementary pair at the interface. Dislocations are positioned as shown in Fig. 1h, and we will refer to dislocation “A” and “B” in the same way. MD simulations (not reported here) showed us that dislocation “B” remains immobile (stuck in a local minimum) until enough vacancies make it climb by a single glide plane. After that, the dislocation glides again, eventually reaching the interface. Notably, the response of a 60$^{\circ }$ misfit dislocation, which is glissile, to the interaction with vacancies is not a pure climb motion. The formation of a 90$^{\circ }$ Lomer at the interface can still be achieved by the motion of two complementary 60$^{\circ }$ dislocations alongside the Si/Ge interface. The situation where two complementary 60$^{\circ }$ misfit dislocations are located nearby at the interface has been revealed in many experiments, in particular before annealing^23,47^. Annealing can cause vacancy migration towards dislocations, making them climb one towards the other, eventually leading to dislocation joining, forming an edge dislocation. To check this hypothesis, we studied the interaction of vacancies with two complementary 60$^{\circ }$ dislocations already at the Si/Ge interface as in Fig. 1k, separated by a distance of ${3}\,\hbox {nm}$. We inserted a vacancy at a time, randomly deleting an atom in a region below the dislocation cores, excluding the cores themselves by the region (excluding a cylindrical area with axis along the *y* direction, a radius equal to ${0.7}\,\hbox {nm}$, centered in the middle point of the two dislocations).

**Figure 4:**
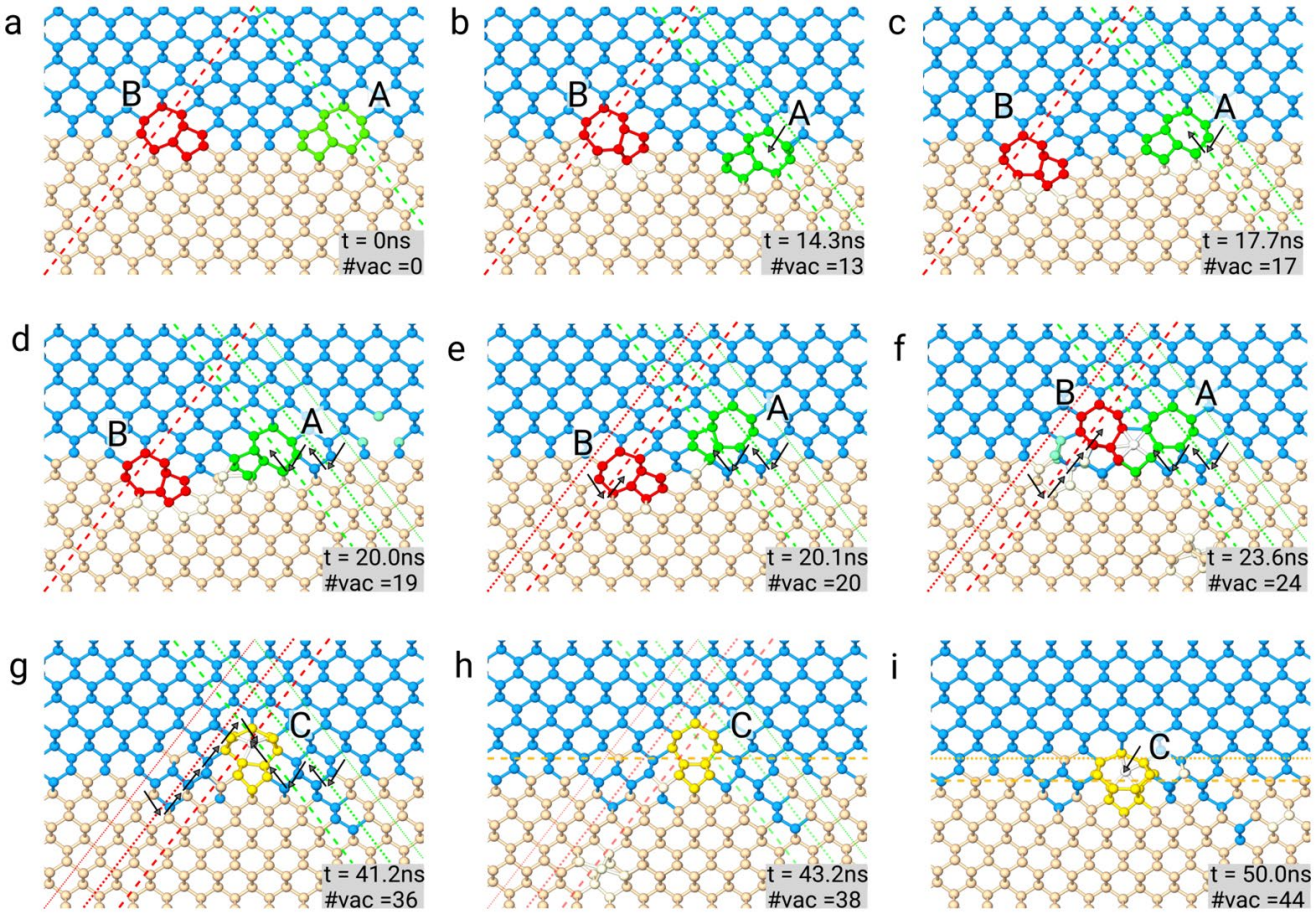
Simulation of a pair of 60$^{\circ }$ dislocations climb. Along all three rows, relevant snapshots of the evolution are shown. Light blue spheres represent Ge atoms, pink spheres Si atoms, green spheres highlight the core of the dislocation “A” (Burgers vector $\vec{b}=a/2[011]$), red spheres highlight the core of the complementary dislocation “B” (Burgers vector $\vec{b}=a/2[{\bar{1}}0{\bar{1}}]$). The glide planes of the dislocations are shown by dashed lines colored accordingly to which dislocation they belong. Simulation snapshots are provided in sequence (**a**–**i**), the number of vacancies inserted in the cell and the corresponding simulation time are shown in the inset. Black arrows show the displacement of the dislocations.

In Fig. 4 we show snapshots of MD simulations of the evolution of vacancies in the presence of the two complementary 60$^{\circ }$ dislocations. In particular, in Fig. 4a the initial position of the two dislocations is shown. The simulated time and the total number of vacancies inserted so far in the cell are shown in the insets. Each dislocation core is highlighted in a different color, we will refer to them following the same convention used in Fig. 1, dislocation “A” for the one with Burgers vector $\vec{b}=a/2[011]$ (colored in green) and dislocation “B” for the one with Burgers vector $\vec{b}=a/2[{\bar{1}}0{\bar{1}}]$ (colored in red). Dashed lines, colored accordingly, also show their glide planes.

After 13 vacancies have been inserted in the cell and a simulated time of more than ${14}\, \hbox {ns}$, dislocation “A” climbs, moving outside its original glide plane (the dotted green line) towards the next glide plane (dashed green line), see Fig. 4b. Now dislocation “A” has climbed inside the Si/Ge interface, and therefore it glides along its glide planes back to the Ge film, moving towards dislocation “B” (Fig. 4c). After that, in the successive two ns, dislocation “A” climbs and glide again, as displayed by the arrows in Fig. 4d. The former glide planes are shown for reference as dotted green lines, with the thinner being the original one. Also dislocation “B” climbs, as shown in Fig. 4e, reducing the distance between the two dislocations. Actually, at such limited distance, the attraction between the two dislocations becomes so intense that dislocation “B” glides along its glide plane and reaches the same height above the interface of dislocation “A.” Still, some more vacancies are needed to make the two dislocations climb and react. Enough vacancies eventually arrive at the dislocation cores after ${41.2}\,\hbox {ns}$, as shown in Fig. 4g (old glide planes are shown by dotted lines for reference). The glide plane of the newly formed 90$^{\circ }$ edge dislocation is 2 mono-layers above the Si/Ge interface (see Fig. 4h). Additional migration of the newly formed 90$^{\circ }$ edge dislocation (which core is highlighted in yellow) happens. It climbs to the Si/Ge interface adding more vacancies in the cell, as shown in the last snapshot (Fig. 4i). The new glide plane is shown by a dashed yellow line, and the former one by a dotted yellow line, an arrow indicates the displacement of the edge dislocation.

In Fig. 4 we have chosen to present a section of the whole-cell taken by slicing the cell perpendicularly to the dislocation lines to ease the view. In effect, there is some disorder at every step of the present simulation because we are simulating the evolution of 60$^{\circ }$ dislocations that are not sessile, as in the case of an edge dislocation. Therefore dislocation segments move easily and not as a whole. As a result, we observed kink formation along the dislocation lines. In order to obtain as final configuration a straight perfect edge dislocation at the interface long as the whole simulation cell, we had to add a 50ns equilibration phase during which the frequency of vacancy insertion was decreased. While not the main aim of this work, we notice how the present simulations, see Fig. 4i, naturally predict the presence of Ge/Si intermixing around dislocation cores and in the surrounding areas, as observed in experiments and justified theoretically based on elastic-energy minimization^23,49^. In such references, the actual microscopic kinetic mechanism was not described. The present simulations seem to show that mixing can be naturally produced by the motion of the vacancies towards dislocation cores. Our simulations also partially reproduced this mixing of Ge atoms in the substrate and vice-versa (note Ge atoms colored in blue in the Si region and vice-versa in Fig. 4f–i).

### Glide of 90$^{\circ }$ misfit dislocation

In the former two sections, we investigated the role of vacancies in dislocation climb. As expected, simulations showed that vacancies are attracted by dislocations, eventually producing their climbing motion. As a result, edge dislocations from the Ge film can migrate to the Si/Ge interface, and pairs of complementary 60$^{\circ }$ dislocations can migrate alongside the interface and there join to form edge dislocations. Based on our results, we can therefore provide the following explanation of the experimental findings^21,29,32^: during low-T growth, a large density of vacancies is introduced in the system as a direct consequence of out-of-equilibrium growth. However, their mobility is limited once again due to the low T. The situation changes when a second stage involving either annealing or high-T growth follows. Vacancies can now quickly diffuse, enabling the interaction of complementary 60$^{\circ }$ misfit dislocation, originally grouped into pairs, eventually forming edge dislocations.

Having provided an answer to the first open question regarding the annealing-induced abundance of 90$^{\circ }$ versus 60$^{\circ }$ dislocations, we can now address the second, i.e., how such edge dislocations can move laterally, producing an ordered distribution as observed experimentally^21,29^.

To answer, we considered the situation where an array of non-equidistant 90$^{\circ }$ is present at the Si/Ge interface. We considered a simulation cell ${20}\,\hbox {nm}$ wide. We inserted two edge dislocations distanced by only ${3}\,\hbox {nm}$ (as we use PBCs, this actually corresponds to an array of dislocations where each defect has a distance of ${3}\,\hbox {nm}$ with the closest defect and of ${17}\,\hbox {nm}$ with the next one) checking whether mechanism leading to an increase of such distance towards the ${10}\,\hbox {nm}$ ideal value was occurring.

A Lomer dislocation has Burgers vector $\vec{b}=a/2[110]$ or $\vec{b}=a/2[{\bar{1}}10]$, and its glide plane is the (001) plane, that coincides with the Ge/Si interface. That plane does not belong to the primary slip system of the zinc-blende/diamond structure. Hence, the glide motion of such a dislocation is not as easy as for 60$^{\circ }$ dislocations. Even if vacancies are not necessarily required for the glide motion of edge dislocations, we will show that their presence is of fundamental importance in accelerating dislocation movement, enabling Lomer dislocation glide on a reduced timescale.

**Figure 5:**
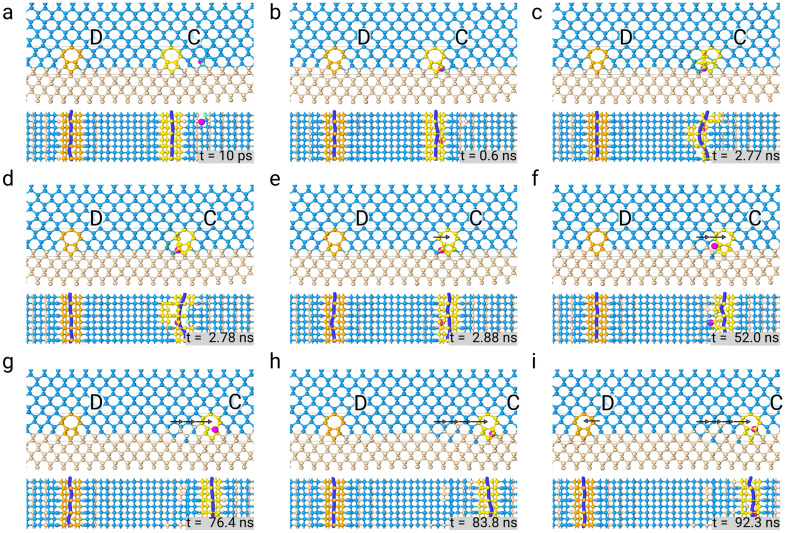
Simulation of 90$^{\circ }$ edge dislocation vacancy-assisted glide. Relevant snapshots of the evolution are shown. Light blue spheres represent Ge atoms, pink spheres Si atoms, yellow spheres highlight the core of the dislocation “C” on the right (Burgers vector $\vec{b}=a/2[{\bar{1}}10]$), orange spheres highlight the core of the identical dislocation “D” on the left (Burgers vector $\vec{b}=a/2[{\bar{1}}10]$). The vacancy, as identified by Wigner–Seitz defect analysis in Ovito, is depicted as a big purple sphere. For each configuration, a top view of a slice around the defects is provided below each snapshot. Thin black lines indicate the sliced region of each snapshot. Simulation snapshots are provided in sequence (**a**–**i**), and the corresponding simulation time is shown in the inset. Black arrows indicate the displacement of the dislocations.

Indeed, we ran an MD simulation at ${1400}\,\hbox {K}$ without any vacancy in the Ge film, and we observed a single gliding event (i.e., lateral motion leading to an increase in lateral distance) after an extremely long simulation time ($\sim {90}\,\hbox {ns}$). At a lower T of ${1200}\,\hbox {K},$ no events at all were witnessed in as long as ${100}\,\hbox {ns}$, the maximum time we reached with our simulations. We conclude that lateral motion can occur but on quite long time scales. Interestingly, however, we noticed that a single vacancy could lead to a noticeable increase in gliding velocity. In Fig. 5, indeed, we show the results of a simulation where a single vacancy is inserted a few lattice sites to the right of Lomer dislocation “C”, in Fig. 5a. The vacancy, represented by a purple sphere, is attracted by the dislocation and reaches its core in a relatively short timescale (Fig. 5b). No vacancy is present near the other edge dislocation “D.” The presence of a vacancy in the dislocation “C” core enables the formation of a kink as shown in Fig. 5c (the top view of the defect, together with the corresponding dislocation line, is provided).

Once the kink has formed, the completion of the glide step is relatively fast; indeed, only $\approx$ ${100}\,\hbox {ps}$ are needed to move the whole dislocation line (see Fig. 5d–e). The vacancy is still around the dislocation core so that it can enhance the glide motion of dislocation “C” again. Indeed, after $\approx$ ${50}\,\hbox {ns}$ dislocation “C” glides on the right again as shown in Fig. 5f (the black arrows are inserted for reference). A third and a fourth glide jumps to the right are observed in the next $\approx$ ${30}\,\hbox {ns}$ (Fig. 5g–h). Notably, we also observe dislocation “D” glide on the left, but after ${92.3}\,\hbox {ns}$ of simulation time. The video animation of the activation of the lateral glide of a 90$^{\circ }$ edge dislocation via vacancy absorption on a three times larger cell along *y* can be found as Supplementary Video S2 and Supplementary Fig. S2 online.

In the presence of the vacancy, a single glide step was also observed in a simulation run at ${1200}\,\hbox {K}$.

## Conclusions

In this work, we exploited molecular dynamics simulations to shed light on the atomic-scale mechanisms leading to the experimentally observed formation of a laterally-ordered array of 90$^{\circ }$ dislocations at the interface of Ge/Si(001) films^21–23,29^. Notably, such arrays are typically obtained in experiments where a first low-T deposition stage is followed by a rise in temperature^21,29^. In the former stage, pairs of 60$^{\circ }$ dislocations are formed by pure gliding motion, which can occur even in the absence of fast-moving vacancies. And, indeed, we could observe the formation of such pairs in MD simulations where no point defects at all were present. Explaining the complete transition of the pairs into 90$^{\circ }$ dislocations required climbing motion to be activated at the higher temperature following the low-T stage. After having introduced a convenient procedure to introduce vacancies in the simulation cell, we were able to observe full vacancy-induced dislocation climbing leading to the transformation of the 60$^{\circ }$ pairs into 90$^{\circ }$ dislocations and/or climbing of 90$^{\circ }$ dislocations at the Ge/Si(001) interface. At this point, we tackled lateral ordering of 90$^{\circ }$ dislocations at the interface. While lateral gliding motion was also observed in the absence of point defects, our simulations revealed how even the presence of a single vacancy could significantly speed up the process, ultimately leading to an ordered array. With this respect, we find it interesting to point out that the final stage of lateral ordering can also be reached without imaging an excess of vacancies in the system, at variance with the transformation of 60$^{\circ }$ into 90$^{\circ }$ in the likely situation where nucleation of the second 60$^{\circ }$ dislocation does not occur on the mirror glide plane.

In conclusion, our simulations reproduced experimental findings and, in doing so, helped in understanding the different phases needed to achieve the limit of a unimodal distribution of laterally-ordered dislocations in Ge/Si(001), highlighting when the role played by vacancies is crucial and when it is not. This can help to devise new growth strategies where alternating close-to-equilibrium and far-from-equilibrium conditions are exploited to influence the dislocation distribution.

## Supplementary Information

Supplementary Video S1.

Supplementary Video S2.

Supplementary Figures.

## References

[1] Verdonckt-Vandebroek S (1994). Sige-channel heterojunction p-mosfet’s. IEEE Trans. Electron Dev..

[2] Schaffler F, Tobben D, Herzog HJ, Abstreiter G, Hollander B (1992). High-electron-mobility si/sige heterostructures: influence of the relaxed sige buffer layer. Semicond. Sci. Technol..

[3] Ismail K, Meyerson BS, Wang PJ (1991). High electron mobility in modulation-doped si/sige. Appl. Phys. Lett..

[4] Engelhardt C (1994). High mobility 2-d hole gases in strained ge channels on si substrates studied by magnetotransport and cyclotron resonance. Solid-State Electron..

[5] Fischetti MV, Laux SE (1996). Band structure, deformation potentials, and carrier mobility in strained si, ge, and sige alloys. J. Appl. Phys..

[6] Erdtmann M, Langdo TA (2006). The crystallographic properties of strained silicon measured by x-ray diffraction. J. Mater. Sci. Mater. Electron..

[7] Franco J, Kaczer B, Groeseneken G (2014). Reliability of High Mobility SiGe Channel MOSFETs for Future CMOS Applications.

[8] Pillarisetty R (2011). Academic and industry research progress in germanium nanodevices. Nature.

[9] Taraschi G, Pitera AJ, Fitzgerald EA (2004). Strained si, sige, and ge on-insulator: review of wafer bonding fabrication techniques. Solid-State Electron..

[10] Liu J, Kimerling LC, Michel J (2012). Monolithic ge-on-si lasers for large-scale electronic–photonic integration. Semicond. Sci. Technol..

[11] Liu J (2014). Monolithically integrated ge-on-si active photonics. Photonics.

[12] yin Hong, C. *et al.* High performance, waveguide integrated ge photodetectors. *Opt. Express* **15**, 3916–3921. 10.1364/OE.15.003916 (2007). 10.1364/oe.15.003916 19532633

[13] Oberhuber R, Zandler G, Vogl P (1998). Subband structure and mobility of two-dimensional holes in strained si/sige mosfet’s. Phys. Rev. B.

[14] Ghani, T. *et al.* A 90 nm high volume manufacturing logic technology featuring novel 45 nm gate length strained silicon CMOS transistors. 11.6.1–11.6.3. 10.1109/IEDM.2003.1269442 (IEEE, 2003).

[15] Sun Y, Thompson SE, Nishida T (2007). Physics of strain effects in semiconductors and metal–oxide–semiconductor field-effect transistors. J. Appl. Phys..

[16] Shchukin V, Bimberg D (1999). Spontaneous ordering of nanostructures on crystal surfaces. Rev. Mod. Phys..

[17] Bergamaschini R, Salvalaglio M, Backofen R, Voigt A, Montalenti F (2016). Continuum modelling of semiconductor heteroepitaxy: an applied perspective. Adv. Phys. X.

[18] Brehm M (2009). Key role of the wetting layer in revealing the hidden path of ge/si(001) Stranski–Krastanow growth onset. Phys. Rev. B.

[19] Zhang J (2010). Collective shape oscillations of sige islands on pit-patterned si(001) substrates: A coherent-growth strategy enabled by self-regulated intermixing. Phys. Rev. Lett..

[20] Montalenti F (2014). Fully coherent growth of ge on free-standing si(001) nanomesas. Phys. Rev. B.

[21] Sakai A, Taoka N, Nakatsuka O, Zaima S, Yasuda Y (2005). Pure-edge dislocation network for strain-relaxed sige/si(001) systems. Appl. Phys. Lett..

[22] Capellini G (2010). Strain relaxation in high ge content sige layers deposited on si. J. Appl. Phys..

[23] Arroyo RD (2019). Effect of thermal annealing on the interface quality of ge/si heterostructures. Scr. Mater..

[24] Dushaq G, Rasras M, Nayfeh A (2017). Low temperature deposition of germanium on silicon using radio frequency plasma enhanced chemical vapor deposition. Thin Solid Films.

[25] Rovaris F, Bergamaschini R, Montalenti F (2016). Modeling the competition between elastic and plastic relaxation in semiconductor heteroepitaxy: From cyclic growth to flat films. Phys. Rev. B.

[26] Kasper, E. & Lyutovich, K. (eds.) *Properties of silicon germanium and SiGe:Carbon* (INSPEC, 2000).

[27] Oktyabrsky S, Wu H, Vispute RD, Narayan J (1995). Misfit dislocations in low-temperature grown ge/si heterostructures. Philos. Mag. A.

[28] Shah V, Dobbie A, Myronov M, Leadley D (2011). High quality relaxed ge layers grown directly on a si(001) substrate. Solid-State Electron..

[29] Kopp VS, Kaganer VM, Capellini G, Seta MD, Zaumseil P (2012). X-ray diffraction study of plastic relaxation in ge-rich sige virtual substrates. Phys. Rev. B.

[30] Bolkhovityanov YB, Deryabin AS, Gutakovskii AK, Sokolov LV (2011). Mechanisms of edge-dislocation formation in strained films of zinc blende and diamond cubic semiconductors epitaxially grown on (001)-oriented substrates. J. Appl. Phys..

[31] Maras E, Pizzagalli L, Ala-Nissila T, Jónsson H (2017). Atomic scale formation mechanism of edge dislocation relieving lattice strain in a gesi overlayer on si(001). Sci. Rep..

[32] Marzegalli A (2013). Onset of plastic relaxation in the growth of ge on si(001) at low temperatures: Atomic-scale microscopy and dislocation modeling. Phys. Rev. B.

[33] Trushin O (2016). Minimum energy path for the nucleation of misfit dislocations in ge/si(0 0 1) heteroepitaxy. Modell. Simul. Mater. Sci. Eng..

[34] Gosling TJ (1993). Mechanism for the formation of $$90^{\circ }$$ dislocations in high-mismatch (100) semiconductor strained-layer systems. J. Appl. Phys..

[35] Kvam EP, Maher DM, Humphreys CJ (1990). Variation of dislocation morphology with strain in $$ge_xsi_{1-x}$$ epilayers on (100)si. J. Mater. Res..

[36] Plimpton S (1995). Fast parallel algorithms for short-range molecular dynamics. J. Comput. Phys..

[37] Tersoff J (1989). Modeling solid-state chemistry: Interatomic potentials for multicomponent systems. Phys. Rev. B.

[38] Marzegalli A, Montalenti F, Miglio L (2005). Stability of shuffle and glide dislocation segments with increasing misfit in si1-xgex/si(001) epitaxial layers. Appl. Phys. Lett..

[39] Hu, S. *et al.* Screw dislocation induced phonon transport suppression in sige superlattices. *Phys. Rev. B*. 10.1103/PhysRevB.100.075432 (2019).

[40] Kumari, S. & Dutta, A. Vacancy-mediated diffusion of atoms at ge/si interfaces: An atomistic perspective. *Materialia*. 10.1016/j.mtla.2020.100666 (2020).

[41] Shinoda W, Shiga M, Mikami M (2004). Rapid estimation of elastic constants by molecular dynamics simulation under constant stress. Phys. Rev. B.

[42] Stukowski A (2009). Visualization and analysis of atomistic simulation data with OVITO-the open visualization tool. Model. Simul. Mater. Sci..

[43] Hirth J, Lothe J (1982). Theory of Dislocations.

[44] Andrews AM, Speck JS, Romanov AE, Bobeth M, Pompe W (2002). Modeling cross-hatch surface morphology in growing mismatched layers. J. Appl. Phys..

[45] López, P., Pelaz, L., Santos, I., Marqués, L. A. & Aboy, M. Molecular dynamics simulations of damage production by thermal spikes in ge. *J. Appl. Phys*. 10.1063/1.3682108 (2012).

[46] Li C, Meng Q (2009). Computer simulation of the vacancy defects interaction with shuffle dislocation in silicon. Superlattices Microstruct..

[47] Zhao C (2016). Strain status of epitaxial ge film on a si (001) substrate. J. Phys. Chem. Solids.

[48] Lanzoni, D., Rovaris, F. & Montalenti, F. Computational analysis of low-energy dislocation configurations in graded layers. *Crystals*. 10.3390/cryst10080661 (2020).

[49] Martinelli L (2004). Formation of strain-induced si-rich and ge-rich nanowires at misfit dislocations in sige: A model supported by photoluminescence data. Appl. Phys. Lett..

