# Supplementary material for: Atomic-scale insights on the formation of ordered arrays of edge dislocations in Ge/Si(001) films via molecular dynamics simulations
Source: Sci Rep. 2022 Feb 25;12:3235. doi: 10.1038/s41598-022-07206-3 (PMC8881629; doi:10.1038/s41598-022-07206-3)
Supplement: Supplementary file 3 — Supplementary Figures. [file 41598_2022_7206_MOESM3_ESM.pdf]

Supplementary Information for:
Atomic-scale Insights on the Formation of Ordered Arrays of Edge Dislocations in Ge/Si(001) Films via Molecular Dynamics Simulations

Luca Barbisan[1,*], **Anna Marzegalli**[2], **and Francesco Montalenti**[1]

[1]L-Ness and Università degli Studi di Milano-Bicocca, Department of Materials Science, Via R. Cozzi 55 Milano, I-20125, Italy
[2]L-Ness and Politecnico di Milano, Department of Physics, via Anzani 42, 22100 Como, Italy
[*]luca.barbisan@unimib.it

Climbing of 90° misfit dislocation

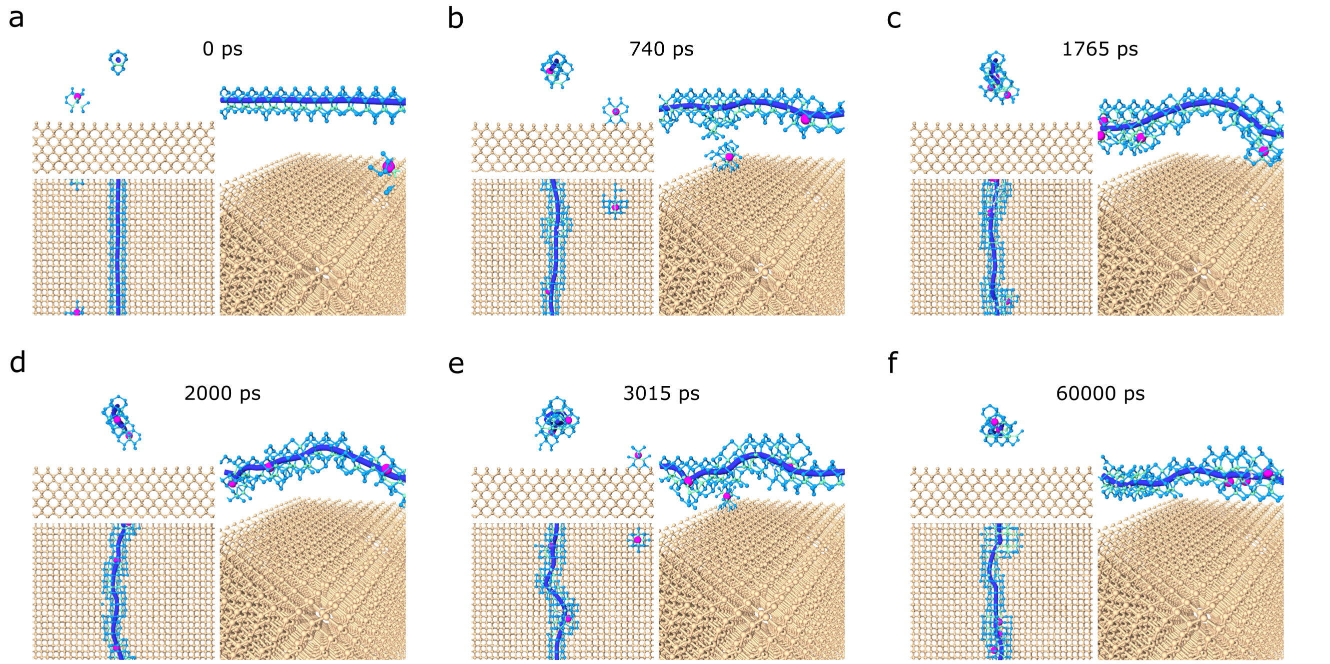

Supplementary Figure S 1. Simulation of edge dislocation climb with a 4.5 nm wide cell, three times larger than the results in the main manuscript. Light blue spheres represent Ge atoms, pink spheres Si atoms, a blue line highlights the core of an edge dislocation 2.0 nm above the interface (Burgers vector $\vec{b} = a/2[\bar{1}10]$). Larger purple spheres show the position of the vacancies as identified by Wigner-Seitz defect analysis in Ovito. Vacancies at the dislocation core since more than 100ps have been hidden. For each configuration, three perspective are shown: side view in the direction of the dislocation line (upper left panel), top view (lower left panel), and front view perpendicular to the dislocation line (right panel). (**a**) Starting configuration: a Lomer dislocation 2.0 nm above the Si/Ge interface. (**b-e**) intermediate steps, the simulation time is shown at the top of each panel. (**f**) final snapshot after 60 ns. The video animation of the simulation is available online as Supplementary Video S1. Vacancy are attracted by the 90° dislocation that moves only upon the arrival of enough vacancies to make each segment climb. Jogs are formed and migrate via the absorption of new vacancies.

Glide of 90° misfit dislocation

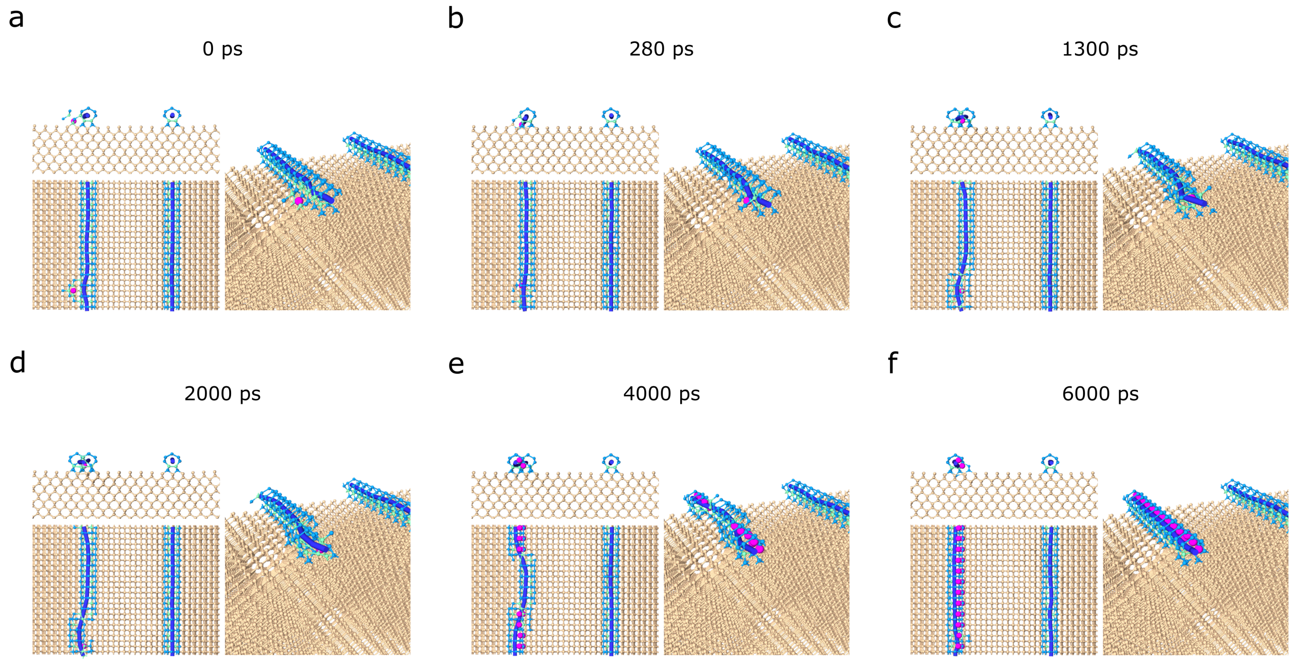

Supplementary Figure S 2. Simulation of 90° edge dislocation vacancy-assisted glide with a 4.5 nm wide cell, three times larger than the results in the main manuscript. Relevant snapshots of the evolution are shown. Light blue spheres represent Ge atoms, pink spheres Si atoms, blue lines highlights the core of the two dislocation (Burgers vector $\vec{b} = a/2[\bar{1}10]$). The vacancy, as identified by Wigner-Seitz defect analysis in Ovito, is depicted as a big purple sphere. For each configuration, three perspective are shown: side view in the direction of the dislocation line (upper left panel), top view (lower left panel), and front view aside the dislocation line (right panel). (**a**) Starting configuration: two Lomer dislocation 3.0 nm one from the other, both at the Si/Ge interface. (**b**-**e**) intermediate steps, the simulation time is shown at the top of each panel. (**f**) final snapshot after 6 ns. The video animation of the simulation is available online as Supplementary Video S2. The vacancy is attracted by the 90° dislocation. Upon the arrival the vacancy the kink formation is activated. The dislocation eventually glides via the migration of the two kinks along the dislocation line. At the end of the glide step, the vacancy is still at the dislocation core, able to trigger again the kink formation. A line of vacancies appears once the dislocation has moved, it is an artifact of the visualization only.
